# Supplementary material for: Heterogeneous firing responses predict diverse couplings to presynaptic activity in mice layer V pyramidal neurons
Source: PLoS Comput Biol. 2017 Apr 14;13(4):e1005452. doi: 10.1371/journal.pcbi.1005452 (PMC5409182; doi:10.1371/journal.pcbi.1005452)
Supplement: S1 Text — (PDF) [file pcbi.1005452.s001.pdf]

Supplementary Material

Heterogenous firing responses leads to diverse coupling to presynaptic activity in mice layer V pyramidal neurons

Y. Zerlaut & A. Destexhe

June 20, 2016

Contents

1 Reduction to the equivalent cylinder 2

2 Mean membrane potential 2

3 Membrane potential response to a synaptic event 4

4 Deriving the power spectrum of the membrane potential fluctuations 9

5 Deriving the fluctuations properties (μ_V, σ_V, τ_V) 10

6 References 11

1 Reduction to the equivalent cylinder

The key of the derivation relies on having the possibility to reduce the complex morphology to an equivalent cylinder (Rall, 1962). We adapted this procedure to capture the change in integrative properties of the membrane that results from the mean synaptic bombardment during active cortical states, reviewed in Destexhe et al. (2003).

For a set of synaptic stimulation $\{\nu_p^e, \nu_i^p, \nu_e^d, \nu_i^d, s\}$, let's introduce the following stationary densities of conductances:

$$\begin{cases} g_{e0}^p = \pi\, d\, \mathcal{D}_e\, \nu_e^p\, \tau_e^p\, Q_e^p \quad ; \quad g_{i0}^p = \pi\, d\, \mathcal{D}_i\, \nu_i^p\, \tau_i^p\, Q_i^p \\ g_{e0}^d = \pi\, d\, \mathcal{D}_e\, \nu_e^d\, \tau_e^d\, Q_e^d \quad ; \quad g_{i0}^d = \pi\, d\, \mathcal{D}_i\, \nu_i^d\, \tau_i^d\, Q_i^d \end{cases} \tag{1}$$

where $\mathcal{D}_e$ and $\mathcal{D}_i$ are the excitatory and inhibitory synaptic densities.

We introduce two activity-dependent electrotonic constants relative to the proximal and distal part respectively:

$$\lambda^p = \sqrt{\frac{r_m}{r_i(1 + r_m g_{e0}^p + r_m g_{i0}^p)}} \quad \lambda^d = \sqrt{\frac{r_m}{r_i(1 + r_m g_{e0}^d + r_m g_{i0}^d)}} \tag{2}$$

For a dendritic tree of total length l, whose proximal part ends at l_p and with B evenly spaced generations of branches, we define the space-dependent electrotonic constant:

$$\lambda(x) = \big(\lambda^p + \mathcal{H}(x - l_p)(\lambda^d - \lambda^p)\big) 2^{-\frac{1}{3} \lfloor \frac{B\,x}{l} \rfloor} \tag{3}$$

where $\lfloor . \rfloor$ is the floor function. Note that $\lambda(x)$ is constant on a given generation, but it decreases from generation to generation because of the decreasing diameter along the dendritic tree. It also depends on the synaptic activity and therefore has a discontinuity at $x = l_p$.

Following Rall (1962), we now define a dimensionless length X:

$$X(x) = \int_0^x \frac{dx}{\lambda(x)} \tag{4}$$

We define $L = X(l)$ and $L_p = X(l_p)$, the total length and proximal part length respectively (capital letters design rescaled quantities).

2 Mean membrane potential

We derive the mean membrane potential $\mu_V(x)$ corresponding to the stationary response to constant densities of conductances given by the means of the synaptic stimulation. We obtain the stationary equations by removing temporal derivatives in Equation, the set of equation governing this mean membrane potential in all branches is therefore:

$$
\begin{cases}
\frac{1}{r_i}\frac{\partial^2 \mu_v}{\partial x^2} = \frac{\mu_v(x) - E_L}{r_m} \\
\qquad - g_{e0}^p\left(\mu_v(x) - E_e\right) - g_{0i}^p\left(\mu_v(x) - E_i\right) \quad \forall x \in [0, l_p] \\
\frac{1}{r_i}\frac{\partial^2 \mu_v}{\partial x^2} = \frac{\mu_v(x) - E_L}{r_m} \\
\qquad - g_{e0}^d\left(\mu_v(x) - E_e\right) - g_{0i}^d\left(\mu_v(x) - E_i\right) \quad \forall x \in [l_p, l] \\
\frac{\partial \mu_v}{\partial x}_{|x=0} = r_i\left(\frac{\mu_v(0) - E_L}{R_m} + G_{i0}^S\left(\mu_v(0) - E_i\right)\right) \\
\mu_v(l_p^-, t) = \mu_v(l_p^+, t) \\
\frac{\partial \mu_v}{\partial x}_{l_p^-} = \frac{\partial \mu_v}{\partial x}_{l_p^+} \\
\frac{\partial \mu_v}{\partial x}_{x=l} = 0
\end{cases}
\tag{5}
$$

Because the reduction to the equivalent cylinder conserves the membrane area and the previous equation only depends on density of currents, the equation governing $\mu_v(x)$ in all branches can be transformed into an equation on an equivalent cylinder of length L. We rescale x by $\lambda(x)$ (see Equation 4) and we obtain the equation verified by $\mu_V(X)$:

$$
\begin{cases}
\frac{\partial^2 \mu_v}{\partial X^2} = \mu_v(X) - v_0^p \quad \forall X \in [0, L_p] \\
\frac{\partial^2 \mu_v}{\partial X^2} = \mu_v(X) - v_0^d \quad \forall X \in [L_p, L] \\
\frac{\partial \mu_v}{\partial X}|_{X=0} = \gamma^p \left(\mu_v(0) - V_0\right) \\
\mu_v(L_p^-) = \mu_v(L_p^+) \\
\frac{\partial \mu_v}{\partial X}_{L_p^-} = \frac{\lambda^p}{\lambda^d}\frac{\partial \mu_v}{\partial X}_{L_p^+} \\
\frac{\partial \mu_v}{\partial X}_{X=L} = 0
\end{cases}
\tag{6}
$$

where:

$$
\begin{aligned}
v_0^p &= \frac{E_L + r_m g_{e0}^p E_e + r_m g_{i0}^p E_i}{1 + r_m g_{e0}^p + r_m g_{i0}^p} \\
v_0^d &= \frac{E_L + r_m g_{e0}^d E_e + r_m g_{i0}^d E_i}{1 + r_m g_{e0}^d + r_m g_{i0}^d} \\
\gamma^p &= \frac{r_i \lambda^p \left(1 + G_i^0 R_m\right)}{R_m} \\
V_0 &= \frac{E_L + G_i^0 R_m E_i}{1 + + G_i^0 R_m}
\end{aligned}
\tag{7}
$$

We write the solution on the form:

$$\begin{cases} \mu_v(X) = v_0^p + A\,\cosh(X) + C\,\sinh(X) \quad \forall\, X \in [0, L_p] \\ \mu_v(X) = v_0^d + B\,\cosh(X-L) + D\,\sinh(X-L) \quad \forall\, X \in [L_p L] \end{cases} \tag{8}$$

- Sealed-end boundary condition at cable end implies $D = 0$
- Somatic boudary condition imply: $C = \gamma^p\,(v_0^p - V_0 + A)$
- Then v continuity imply : $v_0^p + A\,\cosh(L_p) + \gamma^p\,(v_0^p - V_0 + A)\,\sinh(L_p) = v_0^d + B\,\cosh(L_p - L)$
- Then current conservation imply: $A\,\sinh(L_p) + \gamma^p\,(v_0^p - V_0 + A)\,\cosh(L_p) = \frac{\lambda^p}{\lambda^d}\,B\,\sinh(L_p - L)$

We rewrite those condition on a matrix form:

$$\begin{pmatrix} \cosh(L_p) + \gamma^p \sinh(L_p) & -\cosh(L_p - L) \\ \sinh(L_p) + \gamma^p \cosh(L_p) & -\frac{\lambda^p}{\lambda^d}\,\sinh(L_p - L) \end{pmatrix} \cdot \begin{pmatrix} A \\ B \end{pmatrix} = \begin{pmatrix} v_0^d - v_0^p - \gamma^p\,(v_0^p - V_0)\,\sinh(L_p) \\ -\gamma^p\,(v_0^p - V_0)\,\cosh(L_p) \end{pmatrix} \tag{9}$$

And we solved this equation with the `solve_linear_system_LU` method of `sympy`

The coefficients A and B are given by:

$$A = \frac{\alpha}{\beta} \qquad\qquad B = \frac{\gamma}{\delta} \tag{10}$$

where:

$$\begin{aligned}
\alpha &= V_0 \gamma^P \lambda^D \cosh(L_p) \cosh(L - L_p) + V_0 \gamma^P \lambda^P \sinh(L_p) \sinh(L - L_p) \\
&\quad - \gamma^P \lambda^D v_0^d \cosh(L_p) \cosh(L - L_p) - \gamma^P \lambda^P v_0^d \sinh(L_p) \sinh(L - L_p) \\
&\quad - \lambda^P v_0^d \sinh(L - L_p) + \lambda^P v_0^p \sinh(L - L_p) \\
\beta &= \gamma^P \lambda^D \cosh(L_p) \cosh(L - L_p) + \gamma^P \lambda^P \sinh(L_p) \sinh(L - L_p) + \\
&\quad \lambda^D \sinh(L_p) \cosh(L - L_p) + \lambda^P \sinh(L - L_p) \cosh(L_p) \\
\gamma &= \lambda^D \big(V_0 \gamma^P + \gamma^P v_0^d \cosh(L_p) - \gamma^P v_0^d \\
&\quad - \gamma^P v_0^p \cosh(L_p) + v_0^d \sinh(L_p) - v_0^p \sinh(L_p)\big) \\
\delta &= \gamma^P \lambda^D \cosh(L_p) \cosh(L - L_p) + \gamma^P \lambda^P \sinh(L_p) \sinh(L - L_p) \\
&\quad + \lambda^D \sinh(L_p) \cosh(L - L_p) + \lambda^P \sinh(L - L_p) \cosh(L_p)
\end{aligned} \tag{11}$$

3 Membrane potential response to a synaptic event

We now look for the response to $n_{src} = \lfloor \frac{B\,x_{src}}{l} \rfloor$ synaptic events at position x_{src} on all branches of the generation of x_src, those events have a conductance

$g(t)/n_{src}$ and reversal potential E_{rev}. We make the hypothesis that the initial condition correspond to the stationary mean membrane potential $\mu_V(x)$. This potential will also be used to fix the driving force at the synapse to $\mu_v(x_{src}) - E_{rev}$, this linearizes the equation and will allow an analytical treatment. To derive the equation for the response around the mean $\mu_v(x)$, we rewrite Equation 9 in main text with $v(x,t) = \delta v(x,t) + \mu_v(x)$, we obtain the equation for $\delta v(x,t)$:

$$\begin{cases} \frac{1}{r_i}\frac{\partial^2 \delta v}{\partial x^2} = c_m \frac{\partial \delta v}{\partial t} + \frac{\delta v}{r_m}(1 + r_m\, g_{e0}^p + r_m\, g_{i0}^p) \\ \qquad - \delta(x - x_{src})\left(\mu_v(x_{src}) - E_{rev}\right)\frac{g(t)}{n_{src}}, \quad \forall x \in [0, l_p] \\ \frac{1}{r_i}\frac{\partial^2 \delta v}{\partial x^2} = c_m \frac{\partial \delta v}{\partial t} + \frac{\delta v}{r_m}(1 + r_m\, g_{e0}^d + r_m\, g_{i0}^d) \\ \qquad - \delta(x - x_{src})\left(\mu_v(x_{src}) - E_{rev}\right)\frac{g(t)}{n_{src}}, \quad \forall x \in [l_p, l] \\ \frac{1}{r_i}\frac{\partial \delta v}{\partial x}_{|x=0} = C_M \frac{\partial \delta v}{\partial t}_{|x=0} + \frac{\delta v(0,t)}{R_m}(1 + R_m G_{i0}^S) \\ \delta v(l_p^-, t) = \delta v(l_p^+, t) \\ \frac{\partial \delta v}{\partial x}_{l_p^-} = \frac{\partial \delta v}{\partial x}_{l_p^+} \\ \frac{\partial \delta v}{\partial x}_{x=l} = 0 \end{cases} \tag{12}$$

Because this synaptic event is concomitant in all branches at distance x_{src}, we can use again the reduction to the equivalent cylinder (note that the event has now a weight multiplied by n_{src} so that its conductance becomes $g(t)$), we obtain:

$$\begin{cases} \frac{\partial^2 \delta v}{\partial X^2} = \left(\tau_m^p + (\tau_m^d - \tau_m^p)\mathcal{H}(X - L_p)\right)\frac{\partial \delta v}{\partial t} + \delta v \\ \qquad - \left(\mu_v(X_{src}) - E_{rev}\right)\delta(X - X_{src}) \times \\ \qquad\qquad \frac{g(t)}{c_m}\left(\frac{\tau_m^p}{\lambda^p} + (\frac{\tau_m^d}{\lambda^d} - \frac{\tau_m^p}{\lambda^p})\mathcal{H}(X_{src} - L_p)\right) \\ \frac{\partial \delta v}{\partial X}_{|X=0} = \gamma^p\left(\tau_m^S \frac{\partial \delta v}{\partial t}_{|X=0} + \delta v(0,t)\right) \\ \delta v(L_p^-, t) = \delta v(L_p^+, t) \\ \frac{\partial \delta v}{\partial X}_{L_p^-} = \frac{\lambda^p}{\lambda^d}\frac{\partial \delta v}{\partial X}_{L_p^+} \\ \frac{\partial \delta v}{\partial X}_{X=L} = 0 \end{cases} \tag{13}$$

where we have introduced the following time constants:

$$
\begin{aligned}
\tau_m^D &= \frac{r_m\, c_m}{1 + r_m\, g_{e0}^d + r_m\, g_{i0}^d} \\
\tau_m^P &= \frac{r_m\, c_m}{1 + r_m\, g_{e0}^p + r_m\, g_{i0}^p} \\
\tau_m^S &= \frac{R_m\, C_m}{1 + R_m\, G_{i0}^S}
\end{aligned}
\tag{14}
$$

We now use distribution theory (see Appel (2008) for a comprehensive textbook) to translate the synaptic input into boundary conditions at X_{src}, physically this corresponds to: 1) the continuity of the membrane potential and 2) the discontinuity of the current resulting from the synaptic input.

$$
\begin{cases}
\delta v(X_{src}^-, f) = \delta v(X_{src}^+, f) \\
\dfrac{\partial \delta v}{\partial X}_{X_{src}^+} - \dfrac{\partial \delta v}{\partial X}_{X_{src}^-} = -\big(\mu_v(X_{src}) - E_{rev}\big) \times \\
\qquad\qquad \big(\dfrac{\tau_m^p}{\lambda^p} + (\dfrac{\tau_m^d}{\lambda^d} - \dfrac{\tau_m^p}{\lambda^p})\mathcal{H}(X_{src} - L_p)\big)\, \dfrac{g(t)}{c_m}
\end{cases}
\tag{15}
$$

We will solve Equation 13 by using Fourier analysis. We take the following convention for the Fourier transform:

$$
\hat{F}(f) = \int_{\mathbb{R}} F(t)\, e^{-2i\pi f t}\, dt \tag{16}
$$

We Fourier transform the set of Equations 13, we obtain:

$$
\begin{cases}
\dfrac{\partial^2 \hat{\delta v}}{\partial X^2} = \big(\alpha_f^p + (\alpha_f^d - \alpha_f^p)\mathcal{H}(X - L_p)\big)^2\, \hat{\delta v} \\
\dfrac{\partial \hat{\delta v}}{\partial X}_{|X=0} = \gamma_f^p\, \hat{\delta v}(0, f) \\
\hat{\delta v}(X_{src}^-, f) = \hat{\delta v}(X_{src}^+, f) \\
\dfrac{\partial \hat{\delta v}}{\partial X}_{X_{src}^-} = \dfrac{\partial \hat{\delta v}}{\partial X}_{X_{src}^+} - \big(\mu_v(X_{src}) - E_{rev}\big) \times \\
\qquad\qquad \big(r_f^p + (r_f^d - r_f^p)\mathcal{H}(X_{src} - L_p)\big)\, \hat{g(f)} \\
\hat{\delta v}(L_p^-, f) = \hat{\delta v}(L_p^+, f) \\
\dfrac{\partial \hat{\delta v}}{\partial X}_{L_p^-} = \dfrac{\lambda^p}{\lambda^d}\, \dfrac{\partial \hat{\delta v}}{\partial X}_{L_p^+} \\
\dfrac{\partial \hat{\delta v}}{\partial X}_{X=L} = 0
\end{cases}
\tag{17}
$$

where

$$\begin{aligned} \alpha_f^p &= \sqrt{1 + 2i\pi f \tau_m^p} \qquad r_f^p = \frac{\tau_m^p}{c_m \, \lambda^p} \\ \alpha_f^d &= \sqrt{1 + 2i\pi f \tau_m^d} \qquad r_f^d = \frac{\tau_m^d}{c_m \, \lambda^d} \\ \gamma_f^p &= \gamma^p \, (1 + 2i\pi f \tau_m^S) \end{aligned} \tag{18}$$

To obtain the solution, we need to split the solution into two cases:

1. $X_{src} \leq L_p$

   Let's write the solution to this equation as the form (already including the boundary conditions at $X = 0$ and $X = L$):

$$\hat{\delta v}(X, X_{src}, f) = \begin{cases} A_f(X_{src}) \, \big(\cosh(\alpha_f^p \, X) + \gamma^p \, \sinh(\alpha_f^p \, X) \big) \\ \qquad \text{if :} 0 \leq X \leq X_{src} \leq L_p \leq L \\ B_f(X_{src}) \, \cosh(\alpha_f^p \, (X - L_p)) + C_f(X_{src}) \, \sinh(\alpha_f^p \, (X - L_p)) \\ \qquad \text{if :} 0 \leq X_{src} \leq X \leq L_p \leq L \\ D_f(X_{src}) \, \cosh(\alpha_f^d \, (X - L)) \\ \qquad \text{if :} 0 \leq X_{src} \leq L_p \leq X \leq L \end{cases} \tag{19}$$

   We write the 4 conditions correspondingto the conditions in X_{src} and L_p to get A_f, B_f, C_f, D_f. On a matrix form, this gives:

$$M = \begin{pmatrix} \cosh(\alpha_f^p \, X_{src}) + \gamma_f^p \sinh(\alpha_f^p \, X_{src}) & -\cosh(\alpha_f^p \, (X_{src} - L_p)) & -\sinh(\alpha_f^p \, (X_{src} - L_p)) & 0 \\ \alpha_f^p \big(\sinh(\alpha_f^p \, X_{src}) + \gamma_f^p \cosh(\alpha_f^p \, X_{src}) \big) & -\alpha_f^p \sinh(\alpha_f^p \, (X_{src} - L_p)) & -\alpha_f^p \cosh(\alpha_f^p \, (X_{src} - L_p)) & 0 \\ 0 & 1 & 0 & -\cosh(\alpha_f^d \, (L_p - L)) \\ 0 & 0 & \alpha_f^p & -\alpha_f^d \frac{\lambda^p}{\lambda^d} \, \sinh(\alpha_f^d \, (L_p - L)) \end{pmatrix} \tag{20}$$

$$M \cdot \begin{pmatrix} A_f \\ B_f \\ C_f \\ D_f \end{pmatrix} = \begin{pmatrix} 0 \\ -r_f^p I_f \\ 0 \\ 0 \end{pmatrix} \tag{21}$$

   And we will solve it with the `solve_linear_system_LU` method of `sympy`. For the $A_f(X_{src})$ coefficient, we obtain:

$$A_f(X_{src}) = \frac{a_f^1(X_{src})}{a_f^2(X_{src})} \tag{22}$$

   with:

$$
\begin{aligned}
a_f^1(X_{src}) = I_f r_f^P \big(&-\alpha_f^D \lambda^P \cosh\big(L\alpha_f^D - L_p\alpha_f^D - L_p\alpha_f^P + X_s\alpha_f^P\big)\\
&+ \alpha_f^D \lambda^P \cosh\big(L\alpha_f^D - L_p\alpha_f^D + L_p\alpha_f^P - X_s\alpha_f^P\big)\\
&+ \alpha_f^P \lambda^D \cosh\big(L\alpha_f^D - L_p\alpha_f^D - L_p\alpha_f^P + X_s\alpha_f^P\big)\\
&+ \alpha_f^P \lambda^D \cosh\big(L\alpha_f^D - L_p\alpha_f^D + L_p\alpha_f^P - X_s\alpha_f^P\big)\\
a_f^2(X_{src}) = \alpha_f^P \big(&-\alpha_f^D \gamma_f^P \lambda^P \cosh\big(-L\alpha_f^D + L_p\alpha_f^D + L_p\alpha_f^P\big)\\
&+ \alpha_f^D \gamma_f^P \lambda^P \cosh\big(L\alpha_f^D - L_p\alpha_f^D + L_p\alpha_f^P\big) -\\
&\alpha_f^D \lambda^P \sinh\big(-L\alpha_f^D + L_p\alpha_f^D + L_p\alpha_f^P\big)\\
&+ \alpha_f^D \lambda^P \sinh\big(L\alpha_f^D - L_p\alpha_f^D + L_p\alpha_f^P\big)\\
&+ \alpha_f^P \gamma_f^P \lambda^D \cosh\big(-L\alpha_f^D + L_p\alpha_f^D + L_p\alpha_f^P\big)\\
&+ \alpha_f^P \gamma_f^P \lambda^D \cosh\big(L\alpha_f^D - L_p\alpha_f^D + L_p\alpha_f^P\big)\\
&+ \alpha_f^P \lambda^D \sinh\big(-L\alpha_f^D + L_p\alpha_f^D + L_p\alpha_f^P\big)\\
&+ \alpha_f^P \lambda^D \sinh\big(L\alpha_f^D - L_p\alpha_f^D + L_p\alpha_f^P\big)
\end{aligned}
\tag{23}
$$

2. $L_p \leq X_{\text{src}}$

   Let's write the solution to this equation as the form (already including the boundary conditions at $X = 0$ and $X = L$:

$$
\hat{\delta v}(X, X_{src}, f) =
\begin{cases}
E_f(X_{src}) \big(\cosh(\alpha_f^p X) + \gamma^p \sinh(\alpha_f^p X)\big) \\
\qquad \text{if :} 0 \leq X \leq L_p \leq X_{src} \leq L \\
F_f(X_{src}) \cosh(\alpha_f^d (X - L_p)) + G_f(X_{src}) \sinh(\alpha_f^d (X - L_p)) \\
\qquad \text{if :} 0 \leq L_p \leq X \leq X_{src} \leq L \\
H_f(X_{src}) \cosh(\alpha_f^d (X - L)) \\
\qquad \text{if :} 0 \leq L_p \leq X_{src} \leq X \leq L
\end{cases}
\tag{24}
$$

   We write the 4 conditions correspondingto the conditions in X_{src} and L_p to get A_f, B_f, C_f, D_f. On a matrix form, this gives:

   We rewrite this condition on a matrix form:

$$
M_2 = \begin{pmatrix}
\cosh(\alpha_f^p L_p) + \gamma_f^p \sinh(\alpha_f^p L_p) & -1 & 0 & 0 & 0 \\
\alpha_f^p \big(\sinh(\alpha_f^p L_p) + \gamma_f^p \cosh(\alpha_f^p L_p)\big) & 0 & -\alpha_f^d \frac{\lambda^p}{\lambda^d} & 0 \\
0 & \cosh(\alpha_f^d (X_{src} - L_p)) & \sinh(\alpha_f^d (X_{src} - L_p)) & -\cosh(\alpha_f^d (X_{src} - L)) \\
0 & \alpha_f^d \sinh(\alpha_f^d (X_{src} - L_p)) & \alpha_f^d \cosh(\alpha_f^d (X_{src} - L_p)) & -\alpha_f^d \sinh(\alpha_f^d (X_{src} - L))
\end{pmatrix}
\tag{25}
$$

$$M \cdot \begin{pmatrix} E_f \\ F_f \\ G_f \\ H_f \end{pmatrix} = \begin{pmatrix} 0 \\ 0 \\ 0 \\ -r_f^d I_f \end{pmatrix} \tag{26}$$

And we will solve it with the `solve_linear_system_LU` method of `sympy`. For the $E_f(X_{src})$ coefficient, we obtain:

$$E_f(X_{src}) = \frac{e_f^1(X_{src})}{e_f^2(X_{src})} \tag{27}$$

with:

$$\begin{aligned}
e_f^1(X_{src}) &= 2 I_f \lambda^P r_f^D \cosh\left(\alpha_f^D (L - X_s)\right) \\
e_f^2(X_{src}) &= -\alpha_f^D \gamma_f^P \lambda^P \cosh\left(-L\alpha_f^D + L_p\alpha_f^D + L_p\alpha_f^P\right) \\
&+ \alpha_f^D \gamma_f^P \lambda^P \cosh\left(L\alpha_f^D - L_p\alpha_f^D + L_p\alpha_f^P\right) \\
&- \alpha_f^D \lambda^P \sinh\left(-L\alpha_f^D + L_p\alpha_f^D + L_p\alpha_f^P\right) \\
&+ \alpha_f^D \lambda^P \sinh\left(L\alpha_f^D - L_p\alpha_f^D + L_p\alpha_f^P\right) \\
&+ \alpha_f^P \gamma_f^P \lambda^D \cosh\left(-L\alpha_f^D + L_p\alpha_f^D + L_p\alpha_f^P\right) \\
&+ \alpha_f^P \gamma_f^P \lambda^D \cosh\left(L\alpha_f^D - L_p\alpha_f^D + L_p\alpha_f^P\right) \\
&+ \alpha_f^P \lambda^D \sinh\left(-L\alpha_f^D + L_p\alpha_f^D + L_p\alpha_f^P\right) \\
&+ \alpha_f^P \lambda^D \sinh\left(L\alpha_f^D - L_p\alpha_f^D + L_p\alpha_f^P\right)
\end{aligned} \tag{28}$$

From this calculus, we can write the PSP at the soma on the form:

$$\hat{\delta v}(X=0, X_{src}, f) = K_f(X_{src}) \left(\mu_v(X_{src}) - E_{rev}\right) \hat{g(f)} \tag{29}$$

where $K_f(X_{src})$ given by:

$$K_f(X_{src}) = \begin{cases} A_f(X_{src}) & \forall X_{src} \in [0, L_p] \\ E_f(X_{src}) & \forall X_{src} \in [L_p, L] \end{cases} \tag{30}$$

This is obtained by taking a unitary current $I_f = 1$ in the previous calculus.

4 Deriving the power spectrum of the membrane potential fluctuations

The calculus rely on the ability to obtain the power spectrum of the membrane potential fluctuations at the soma $P_V(f)$.

This can be obtained from shotnoise theory (Daley and Vere-Jones, 2007) (see also El Boustani et al. (2009) for an application similar to ours), the general form of the power spectrum density can be expressed as:

$$P_V(f) = \sum_{\{syn\}} N_{syn}\, F_{\text{synch}}\, \nu_{syn}\, \|\hat{\text{PSP}}_{syn}(f)\|^2 \tag{31}$$

where $\{syn\}$ is the set of identical synapses, each having N_{syn} synapses, a Poisson release probability: ν_{syn} and creating a post-synaptic event $\text{PSP}_{syn}(t)$. In addition, F_{synch} is a synchrony factor (depending on the variable s in the model), it accounts for the effects of the synchronous arrivals of presynaptic events. Given the synchrony generator considered in the main text, the synchrony factor takes the form:

$$F_{\text{synch}} = (1-s) + (s-s^2)2^2 + (s^2-s^3)3^2 + s^3 4^2 \tag{32}$$

because single events arise with a probability $1-s$, double events with a probability $s-s^2$ (and the PSP are squared in Eq. 4, hence the 2^2 factor), etc...

Now obtaining the power spectrum density $P_V(f)$ in our situation requires to explicit the sum over synapses: $\sum_{\{syn\}}$, In our cases, we need to sum over 1) their type (excitatory/inhibitory, $\sum_{s \in \{e,i\}}$), 2) their location (we will integrate over the dendritic length $\int_0^L dx$) 3) branches.

$$\begin{aligned} P_V(f) = \sum_{s \in \{e,i\}} \int_0^L & dx\, \pi\, \mathcal{D}_s \Big(d_t\, 2^{-\frac{2}{3} \lfloor \frac{B\,x}{l} \rfloor} \Big)\; 2^{\lfloor \frac{B\,x}{l} \rfloor}\;\; F_{\text{synch}}\, \nu_s(x)\, \|\hat{\delta v}_s(0,x,f)\|^2 \\ & + \pi\, \mathcal{D}_i\, l_S\, d_S\;\; F_{\text{synch}}\, \nu_i(0)\, \|\hat{\delta v}_i(0,0,f)\|^2 \end{aligned} \tag{33}$$

where $\hat{\delta v}_s(0,x,f)$ is given by Eq. 29 (note that the dependency on synaptic type s comes from the reversal potential term E_{rev} in Eq. 29). The factor $2^{\lfloor \frac{B\,x}{l} \rfloor}$ corresponds to the sum of the synapses over the different branches at the distance x. The term $\big(d_t\, 2^{-\frac{2}{3} \lfloor \frac{B\,x}{l} \rfloor} \big)$ is the diameter of the branches at the distance x.

The last term in Eq. 33 corresponds to the contribution of somatic inhibitory synapses (number of somatic inhibitory synapses: $\pi\, \mathcal{D}_i\, l_S\, d_S$.

5 Deriving the fluctuations properties $(\mu_V, \sigma_V, \tau_V)$

The final expressions for the fluctuation propeorties as a function of $(\nu_e^p, \nu_i^p, \nu_e^d, \nu_i^d, s)$ are thus given by:

- μ_V: we obtain the mean of the fluctuations at the soma by taking $\mu_V(0)$ in Equation 8.

- σ_V: we obtain the standard deviation of the fluctuations from the power spectrum density in Equation 33 and the expression:

$$\sigma_V^2 = \int_{\mathbb{R}} P_V(f)\, df \tag{34}$$

  This integral expression was discretized and evaluated numerically

- τ_V: we obtain the autocorrelation time of the fluctuations from the power spectrum density in Equation 33 and the expression (Zerlaut et al., 2016):

$$\tau_V = \frac{1}{2} \Big(\frac{\int_{\mathbb{R}} P_V(f)\, df}{P_V(0)} \Big)^{-1} \tag{35}$$

  This integral expression was discretized and evaluated numerically

6 References

Appel W (2008) *Mathématiques pour la physique et les physiciens* H. et K. Editions.

Daley DJ, Vere-Jones D (2007) *An introduction to the theory of point processes: volume II: general theory and structure*, Vol. 2 Springer Science & Business Media.

Destexhe A, Rudolph M, Paré D (2003) The high-conductance state of neocortical neurons in vivo. *Nature reviews. Neuroscience* 4:739–751.

El Boustani S, Marre O, Béhuret S, Baudot P, Yger P, Bal T, Destexhe A, Frégnac Y (2009) Network-state modulation of power-law frequency-scaling in visual cortical neurons. *PLoS computational biology* 5:e1000519.

Rall W (1962) Electrophysiology of a dendritic neuron model. *Biophysical journal* 2:145.

Zerlaut Y, Telenczuk B, Deleuze C, Bal T, Ouanounou G, Destexhe A (2016) Heterogeneous firing response of mice layer V pyramidal neurons in the fluctuation-driven regime. *The Journal of Physiology* in press.
