# Supplementary figures and images for: Heterogeneous firing responses predict diverse couplings to presynaptic activity in mice layer V pyramidal neurons
Source: PLoS Comput Biol. 2017 Apr 14;13(4):e1005452. doi: 10.1371/journal.pcbi.1005452 (PMC5409182; doi:10.1371/journal.pcbi.1005452)

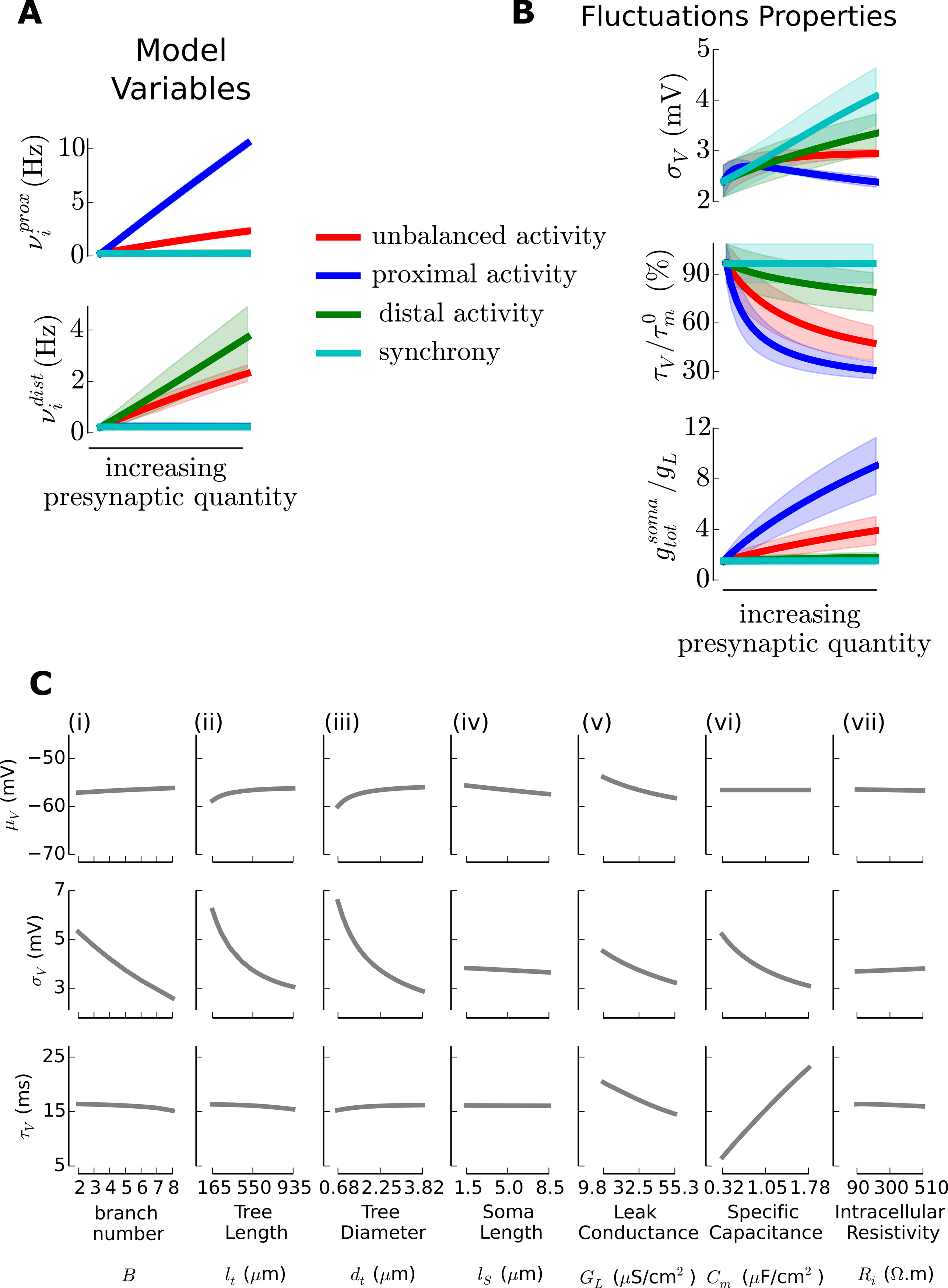

Supplement: S2 Fig — (A) Variability in the model variables across protocols introduced by the different morphologies (sizes). We show only the quantities that vary across cells, all other variables (νep,νed,s) are fixed across cells. The balance μV is adjusted for each cells and the cells have different surfaces, so different number of synapses (and especially different ratio of excitatory to inhibitory numbers) hence the need to adjust inhibitory activity slightly differently for each cell. (B) Variability in the properties of the membrane potential fluctuations (standard deviation σV and autocorrelation time τV) across protocols introduced by the different morphologies (sizes). We show only the quantities that vary across cells, μV is fixed across cells by design. (C) Dependency of the fluctuations properties as a function of the morphology parameters (all parameters are varied of -70% and +70% around those of the mean model, one parameter is varied while all others are fixed to those of the mean model, see Table 1). We fixed the presynaptic stimulation to a level of νep=νed=0.3Hz, νip=νid=1.7Hz and s = 0.05. Globally, the mean polarization μV is poorly affected by the morphology, given the balanced nature of the input and the homogenous spread of synapses on the membrane. On the other hand, the morphology parameters strongly affect the properties of the amplitude and speed of the fluctuations. (i) Dependency on the branching number. Because the branching number has an impact on the area, the number of synapses increases with the number of branches, then the amplitude σV of the fluctuations strongly decreases with the number of branches because of the law of large numbers. (ii) Dependency on the tree length. The same argument as in (i) holds. The length of the tree increases the number of synapses so that it reduces the amplitude of the fluctuations. (iii) Dependency on the tree diameter. The same argument as in (i) holds again. The diameter of the tree increases the number of synaps [file pcbi.1005452.s003.tiff]

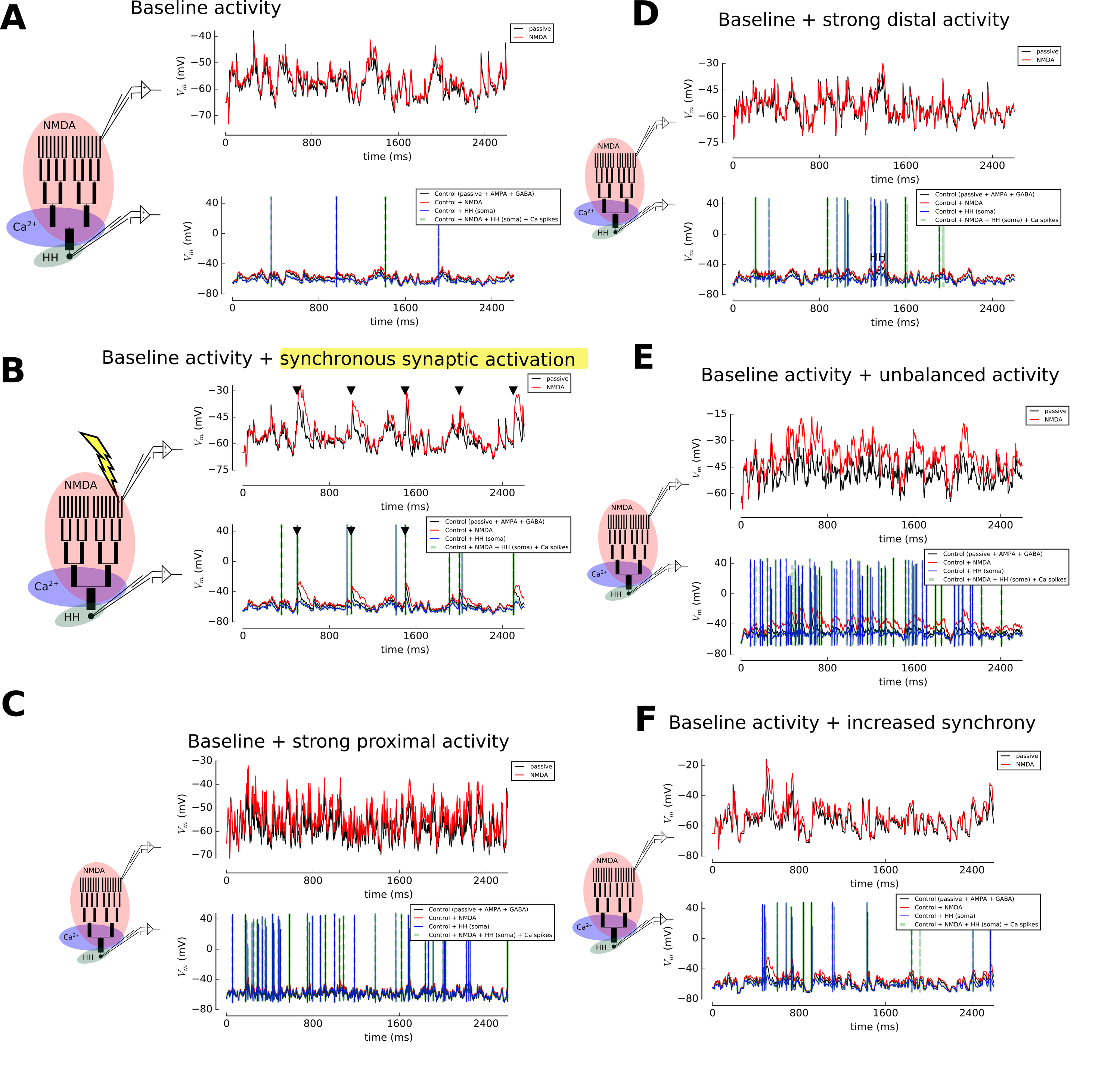

Supplement: S4 Fig — Active mechanisms are taken from Larkum et al. [31]. On top of each panel, we present the recording of the membrane potential in the far distal dendrite (depicted with the upper electrode on the drawing) in the Control and Control+NMDA case. On the bottom, we present the membrane potential recorded at the soma (depicted with the lower electrode on the drawing) in four cases, the Control, the Control+NMDA case, the Control case with a HH model inserted in the soma and the Control+NMDA+Ca2+spikes+HH(soma). (A) Response to baseline activity. νep=νed = 0.2Hz, νip=νid = 1.2Hz and s = 0.05. (B) Response to baseline activity with the synchronous activation of 20 synapses every 500ms (marked by a triangle), note the impact of the NMDA mechanism under this stimulation type. (C) Response to an increase of proximal activity with respect to the baseline level (νep = 1.6Hz, νip = 10Hz). (D) Response to an increase in distally targeting presynaptic activity (νed = 0.6Hz, νid = 3Hz). (E) Response to an increase in unbalanced activity (νep = 0.4Hz, νip = 2Hz, νep = 0.5Hz, νip = 1.7Hz). (F) Response to an increase in presynaptic synchrony (s = 0.4). (TIFF) [file pcbi.1005452.s005.tiff]

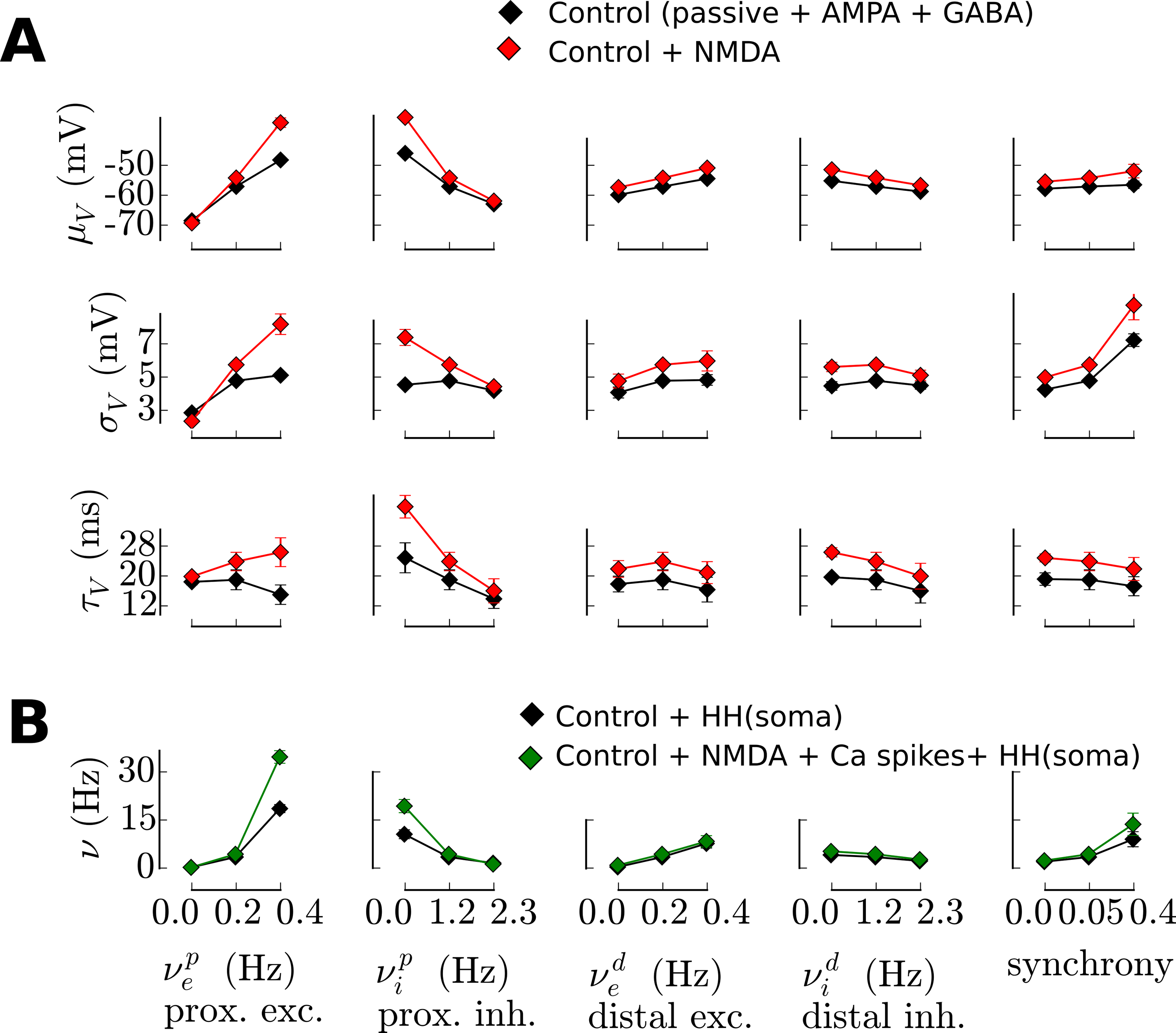

Supplement: S5 Fig — Because our framework for single cell computation consists in (i) evaluating analytically the somatic subthreshold membrane potential fluctuations at the soma as a function of the presynaptic quantities and (ii) convert those fluctuations into a spiking probability thanks to a firing response function determined in vitro in individual neuron, it needs to be tested against two phenomena: (I) whether active dendritic mechanisms qualitatively affect the relationship between presynaptic quantities and somatic membrane potential fluctuations, i.e. checking the validity of step (i), and (II) whether dendritic non-linearities introduce a coupling between presynaptic activity and spike emission that renders the decomposition into the successive steps i & ii problematic. Those phenomena will naturally appear to some extent and will produce quantitative deviations with respect to our predictions (that do not include dendritic non-linearities, e.g. trivially, all cells should be more excitable). As our results do not rely on the absolute values of the input-output functions but rather on the relative behaviors from cell to cell (that emerge from differences across individual firing response functions), we only look for putative "qualitative deviations". (A) We first investigate whether active dendritic mechanisms qualitatively affect the relationship between presynaptic quantities and somatic membrane potential fluctuations. We observe that NMDA channels exert an additional small depolarization (increased μV), they also increase the standard deviation of the fluctuations because they increase the amplitude of depolarizing events (increased σV) and they slow down the fluctuations because they increase the low frequency content of post- synaptic depolarization (increased τV). We also observe, that, despite this small shift in all quantities, the qualitative trend of all curves remains identical. Only for high excitation, a qualitative difference can be seen (see bottom left plot [file pcbi.1005452.s006.tiff]
